# A comparison of the characteristics and treatment outcomes of migrant and Australian-born users of a national digital mental health service

**DOI:** 10.1186/s12888-020-02486-3

**Published:** 2020-03-11

**Authors:** Rony Kayrouz, Eyal Karin, Lauren G. Staples, Olav Nielssen, Blake F. Dear, Nickolai Titov

**Affiliations:** 1 MindSpot Clinic, Macquarie University, Sydney, Australia; 2 eCentreClinic, Department of Psychology, Macquarie University, Sydney, New South Wales 2109 Australia

**Keywords:** Migrants, Digital mental health service, Online treatment, Anxiety, Depression, iCBT, Ethnicity

## Abstract

**Background:**

To explore the characteristics and compare clinical outcomes of non-Australian born (migrant) and Australian-born users of an Australian national digital mental health service.

**Methods:**

The characteristics and treatment outcomes of patients who completed online treatment at the MindSpot Clinic between January 2014 and December 2016 and reported a country of birth other than Australia were compared to Australian-born users. Data about the main language spoken at home were used to create distinct groups. Changes in symptoms of depression and anxiety were measured using the Patient Health Questionnaire-9 Item (PHQ-9), and Generalized Anxiety Disorder Scale – 7 Item (GAD-7), respectively.

**Results:**

Of 52,020 people who started assessment at MindSpot between 1st January 2014 and 22nd December 2016, 45,082 reported a country of birth, of whom 78.6% (*n* = 35,240) were Australian-born, and 21.4% (*n* = 9842) were born overseas. Of 6782 people who completed the online treatment and reported country of birth and main language spoken at home, 1631 (24%) were migrants, 960 (59%) were from English-speaking countries, and 671 (41%) were from non-English speaking countries. Treatment-seeking migrant users reported higher rates of tertiary education than Australian-born users. The baseline symptom severity, and rates of symptom reduction and remission following online treatment were similar across groups.

**Conclusions:**

Online treatment was associated with significant reductions in anxiety and depression in migrants of both English speaking and non-English speaking backgrounds, with outcomes similar to those obtained by Australian-born patients. DMHS have considerable potential to help reduce barriers to mental health care for migrants.

## Background

Australia has the third highest proportion of residents born overseas of the OECD countries, with around 28% of the population born elsewhere [1]. In 2016, the five countries providing the most migrants were England (14.7%), New Zealand (8.4%), China (8.3%), India (7·4%) and the Philippines (3.8%) [2], reflecting two broad groups of migrants, those of English-speaking background (ESB) and those from Non-English-speaking background countries (NESB). Migration to most countries is associated with an increased likelihood of having or developing a mental illness [3]. However, the 2007 National Survey of Mental Health and Wellbeing survey found that the overall rates of anxiety and mood disorders in Australians born overseas were lower than the Australian-born population, although rates were higher among some refugees [4].

Equity of access to mental health services is a priority for most governments. However research shows that migrants, especially those of NESB, experience barriers to accessing treatment, including low mental health literacy, a lack of suitable services and a lack of trust of services [5], as well as the more universal barriers of stigma and cost [6]. In Australia, there is emerging evidence of increasing uptake of face-to-face treatment by migrants [7], but despite this trend, the use of mental health services by many migrant groups remains low.

An important development in the effort to increase access to mental health care for common mental disorders has been the introduction of digital mental health services (DMHS), that is, treatment delivered via the internet. A large number of clinical trials have demonstrated the efficacy of internet-delivered cognitive behaviour therapy (iCBT) for anxiety and depression [8], with some trials indicating encouraging outcomes when interventions are translated into other languages (e.g., [9, 10]). Furthermore, studies have shown that psychological treatments are just as effective in non-Western countries as they are in Europe and Anglophone countries [11].

iCBT is now available in several countries as part of routine care, and when administered in a systematic way, can achieve large clinical improvements that match those of clinical trials [12]. One of these services is the MindSpot Clinic, established as part of the Australian Government’s eMental Health strategy in order to improve the availability of mental health services for adults with anxiety and depression, particularly for people who may not access face-to-face care [13]. MindSpot (www.mindspot.org.au) provides free assessment and treatment, offering seven iCBT treatment courses, including the Wellbeing Course [14, 15]. The Wellbeing Course is a transdiagnostic treatment that targets core symptoms of anxiety and depression, is designed for adults aged 26–65 years, and is associated with improvements in symptoms of anxiety and depression of around 50%, and deterioration rates of 2% [13, 16, 17]. Outcomes for Indigenous Australians are equivalent to those of non-Indigenous Australians [18]. These encouraging results have influenced the Australian Government Productivity Commission’s draft report on mental health recommending the expansion of supported online mental health treatment to cater for migrants [19]. However, to date, no studies have examined outcomes for migrants from iCBT delivered in routine care.

The aims of the present study were (1) to compare the characteristics of those who started treatment at MindSpot and identified a country of birth other than Australia (i.e., migrants) with Australian-born patients and (2) to compare the treatment outcomes of migrants and Australian-born patients.

## Methods

The study employed a retrospective uncontrolled observational cohort study design, following the recommended STROBE methodological checklist for observational cohort studies [20].

### Participants

Interested adults completed an online screening assessment through the MindSpot website (www.mindspot.org.au), which provides information about common mental health disorders and treatment of these disorders. Inclusion criteria for MindSpot were: (1) Adults aged at least 18 years; (2) currently living in Australia; and (3) were eligible for treatment under Medicare, the Australian national health insurance program. Inclusion criteria for this current study were: (1) reported county of birth; (2) reported main language spoken at home; and (3) enrolled in the Wellbeing Course.

**Fig. 1:**
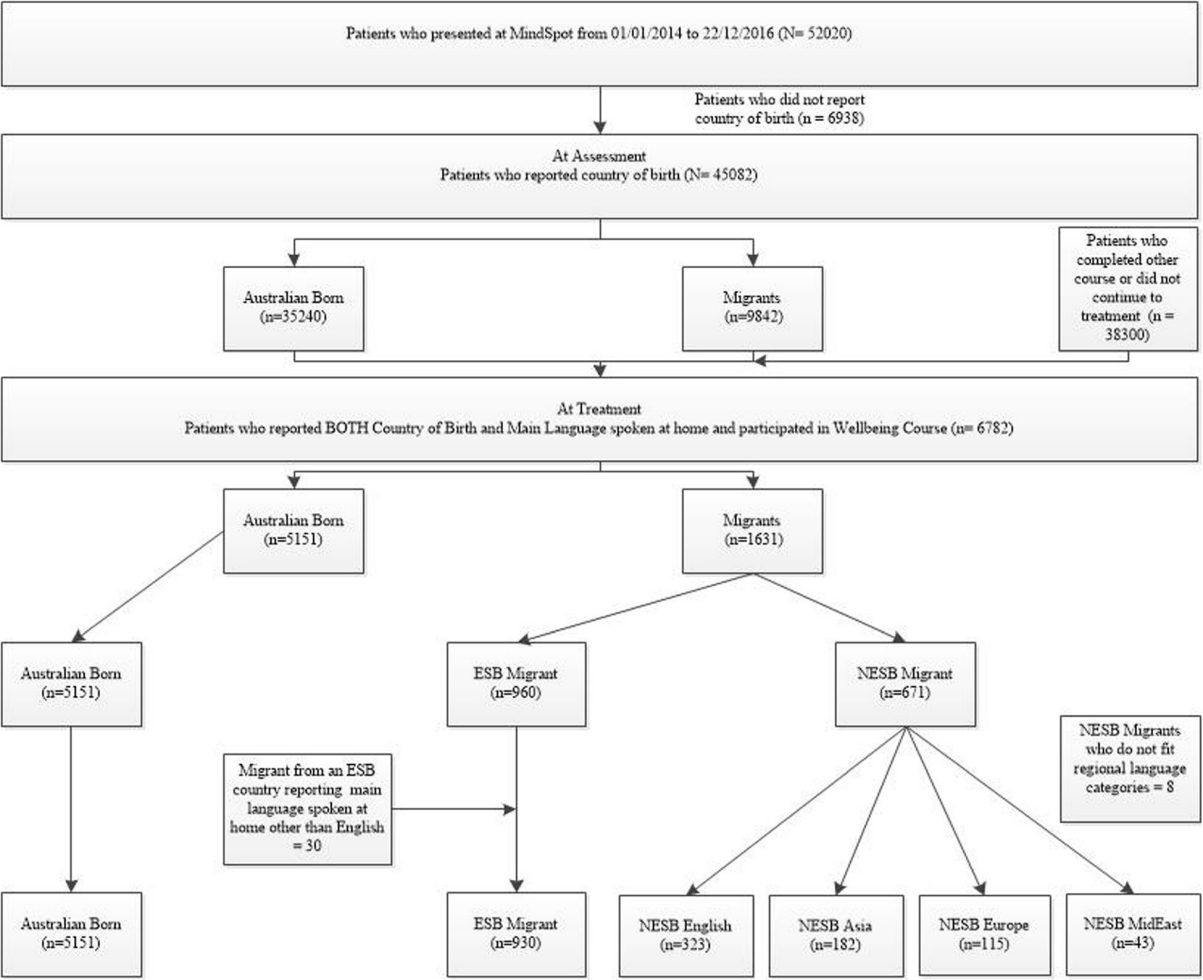
Sample sizes of patients at assessment and treatment. Note. ESB Migrant = Migrant of an English Speaking Background (e,g., England, New Zealand, United States of America etc); NESB Asia = Migrant from the Asian Region (e.g., China, Malaysia, India etc); NESB Europe = Migrants from the Europe region (e.g., Italy, Spain, Greece, Germany etc); NESB MidEast = Migrant from the Middle Eastern or North Africa Region (e.g., Lebanon, Iraq, Egypt etc),; NESB Migrant = Migrant of a Non-English Speaking Background

Between the 1 January 2014 and 22 December 2016, 52,020 people completed a MindSpot assessment, of whom 45,082 reported a country of birth. Of these 78.6% (*n* = 35,240) were Australian-born, and 21.4% (*n* = 9842) were migrants, from 166 countries and jurisdictions. Of the 45,082, 15% (6782) enrolled for treatment with the online Wellbeing Course (see Fig. 1). Patients provided demographic details at the time of registration, including country of birth and, if other than Australia, the main language spoken at home, and completed a series of validated symptom questionnaires relevant to their presenting problems. The Wellbeing Course was only available in English and was delivered over eight weeks, and is described in detail elsewhere. More detail about the assessment procedures, treatment courses, and the methods of maintaining patient safety are described elsewhere [13, 21]. The MindSpot service was provided at no cost to participants. All participants provided consent for their data to be used for research purposes via an online form, which they are presented with at their first engagement with the service. Approval to conduct the study was obtained from the Macquarie University Human Research Ethics Committee.

**Fig. 2:**
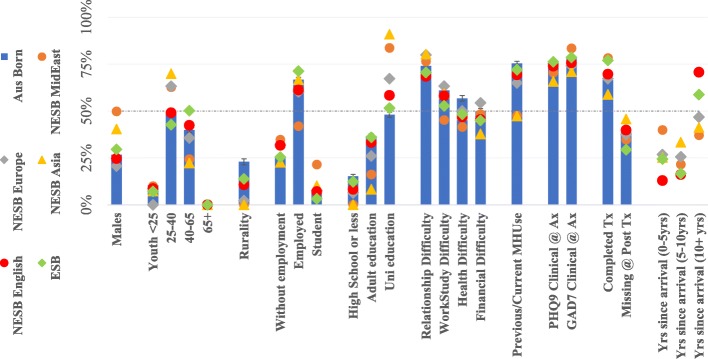
Characteristics of Cohorts at Treatment**.** Note*.* Aus = Australian; Ax = Assessment; ESB = English Speaking Background; Europe = Europe region; GAD7 = Generalised Anxiety Disorder Scale (7-item); MHuse = mental health service use; MidEast = Middle Eastern or North African Region; NESB = Non-English Speaking Background; PHQ9 = Patient Health Questionnaire (9-item); Tx = Treatment; yrs. = years

Information about the demographic characteristics, previous service use, clinical symptoms and additional language and migration features of the treatment sample, are further detailed in Table 1, illustrated graphically in Fig. 2 (the sample who continued to participate in Wellbeing Course).

**Table 1:** Demographic Characteristics, Symptom Scores and Mental Health Service Use of the Cohorts at Treatment

	Migrants by Language Regions													
	AusBorn		NESB MidEast		NESB Europe		NESB Asia		NESB English		ESB English		*P*-value from Test of cohort differences with adjusted Bootstrapping function*	Nagelkerke R Square associated with cohort differences
n	5151	74%	43	1%	115	2%	182	3%	323	5%	930	13%		
Males	1365	27%	22	51%	27	23%	70	38%	79	25%	270	29%	.007	0.2%
18–24	527	10%	3	7%	1	1%	11	6%	22	7%	46	5%	.016	0.7%
25–40	2567	50%	27	63%	73	63%	128	70%	162	50%	394	42%	*p* < .001	0.8%
40–65	2053	40%	13	30%	41	36%	43	24%	138	43%	490	53%	*p* < .001	1.0%
Rural Location	1181	23%	0	0%	8	7%	2	1%	38	12%	154	17%	*p* <. 001	2.2%
Unemployed	1307	25%	17	40%	38	33%	43	24%	94	29%	230	25%	.10	0.2%
Employed	3442	67%	18	42%	68	59%	121	67%	207	64%	667	72%	.001	0.4%
Student	402	8%	8	19%	9	8%	17	9%	22	7%	33	4%	*p* < .001	1.1%
High School or less	784	15%	0	0%	8	7%	2	1%	26	8%	100	11%	*p* < .001	2.2%
Adult Education	1884	37%	6	14%	33	29%	19	10%	104	32%	346	37%	*p* < .001	1.6%
University Education	2483	48%	37	86%	74	64%	161	88%	193	60%	484	52%	*p* < .001	3.4%
Work/Study Difficulties	3843	75%	34	78%	94	82%	151	83%	241	74%	721	78%	.059	0.3%
Relationship Difficulties	3187	62%	24	56%	81	70%	123	67%	215	66%	593	64%	*p* < .001	0.2%
Health Difficulties	2968	58%	18	41%	63	55%	98	54%	166	51%	518	56%	.13	0.1%
Financial Difficulties	2602	51%	23	55%	67	59%	79	43%	162	50%	492	53%	.31	0.1%
Mental Health Service Use	3876	75%	19	44%	77	67%	89	49%	224	69%	694	75%	*p* < .001	1.4%
Clinical levels of PHQ9 symptoms	3796	74%	28	65%	84	73%	120	66%	236	73%	699	75%	.16	0.1%
Clinical levels of GAD7 symptoms	4063	79%	34	79%	84	73%	126	69%	236	73%	728	78%	.006	0.0%
Treatment Completion Rate	67%	3852	78%	31	67%	68	59%	72	70%	190	77%	530	*p* < .001	0.8%
Average lesson completion (of 5)	3.86		3.88		3.76		3.58		3.96		4.06		*p* < .001	0.4%
Missing cases at Post-treatment	1943	38%	18	42%	44	38%	76	42%	117	36%	283	30%	*p* < .001	0.1%
Years since arrival to Australia (0-5 yrs)	–	–	16	37%	31	27%	37	21%	38	12%	221	24%	*p* < .001	3.3%
Years since arrival (5-10 yrs)	–	–	9	21%	29	25%	65	36%	55	17%	147	16%	*p* < .001	2.8%
Years since arrival (10+ yrs)	–	–	18	42%	55	48%	80	44%	230	71%	562	60%	*p* < .001	4.8%

Note*. Aus* Australian, *Ax* Assessment, *ESB* English Speaking Background, *Europe* Europe region, *GAD7* Generalized Anxiety Disorder Scale (7-item), *MidEast* Middle Eastern or North Africa Language Region, *n* cohort sample size *NESB* Non-English Speaking Background, *PHQ9* Patient Health Questionnaire (9-item), *Tx* Treatment, *yrs*. years

### Design

The clinical outcomes of migrant and Australian-born groups in treatment were explored in a series of steps, (1) identifying cohorts of Australian-born, NESB and ESB migrants, (2) the comparison of demographic characteristics, symptom scores and past and current service use, and (3) the comparison of clinical trajectory of these cohorts at post-treatment.

The classification of migrant cohorts was based on the Australian Bureau of Statistics classification of migrants in the Australian community [22, 23]. The final classification employed in this study involved two steps, resulting in a model that attempted a compromise between reflecting cultural and linguistic diversity and obtaining appropriate sample size.

In the first step, migrants were classified as being either from an English-speaking background (ESB) or non-English-Speaking background (NESB) depending on whether English was one of the official languages of the country of origin (see Additional file 1 for details). A second step examined the effect of the main language spoken at home, divided into the following five groups; (1) English, (2) South or East Asian language, (3) Arabic or other middle-eastern language, (4) a European language or (5) other than the above (see Additional file 2 for details).

These two steps (country of origin and language spoken at home)) were used to form the following six distinct cohorts of online treatment users: (1) NESB Asian (South or East Asian regions; Asian language; *n* = 182); (2) NESB MidEast (Middle East or North Africa; Middle Eastern or Arabic language; *n* = 43); (3) NESB Europe (Europe; European language; *n* = 115); (4) ESB (migrants from countries where the national language was English; *n* = 930) and ESB migrants with a main language spoken at home other than English (*n* = 30) were excluded (see Fig. 1 and Additional file 3); (5) NESB English (migrants born in countries where the main language was not English, but English was the main language spoken at home; *n* = 323); and (6) AusBorn (people born in Australia; *n* = 5028). Australian born participants whose main language spoken at home was not English (*n* = 123, 2%) were also assumed to be native English speakers and were included in the analysis (*n* = 5151). NESB migrants who did not fit into the regional language categories (*n* = 8) were excluded (see Fig. 1 and Additional file 3).

Finally, sensitivity analyses were conducted to examine; (1) the duration of residence in Australia; (2) the potential effect of outcome measurement bias associated with missing cases; and (3) the effect of differences in demographic, other service use or baseline clinical characteristics identified between the treatment cohorts.

### Measures

The clinical outcomes of the treatment cohorts were determined using a measure of depression symptoms (Patient Health Questionnaire - 9 item; PHQ-9; [24]) and anxiety symptoms (Generalized Anxiety Disorder – 7 Item; GAD-7; [25]). These measures were administered at assessment, weekly during treatment, and at post treatment to examine the treatment effectiveness as measured by a reduction in symptoms (pre-post). The Cronbach’s alphas for PHQ-9 and GAD-7 in the present study (α = .73 and α = .71 respectively) were considered to be acceptable.

Each week patients completed the GAD-7, PHQ-9 and single-item measures enquiring about personal safety and treatment satisfaction. The GAD-7 and PHQ-9 were administered at assessment and post-treatment, that is, eight weeks after the starting the online treatment course. Questionnaires were self-report measures administered online. These questionnaires appeared automatically after login each week and again at post-treatment. Patient data was collected in an electronic database. Completion of a treatment course was defined as reading the first four lessons of the Wellbeing Course. All participants who started treatment were analysed at post-treatment regardless of whether they had completed the lessons (i.e., refer to the missing cases analyses in the assessment of measurement bias of the analytic plan section).

### Analytic plan

#### Statistical methods

Statistical analyses were used to estimate and compare (1) the characteristics of the cohorts that started treatment, (2) the longitudinal symptom change in each cohort during treatment, (3) the probability of events such as symptom deterioration, no response, minimal response and remission.

The exploration of the treatment sample was operationalised with a series of binary logistic regressions that test the prevalence of different demographic, clinical, service use, language and migration characteristics in each of the five migrant cohorts and the AusBorn group (used as the reference group). These regression models also employed an adjusted bootstrapping procedure to account for differences in the size of each cohort [26].

To compare the efficacy of treatment within each of the cohorts, longitudinal generalised estimation equation (GEE) models [27] were employed, to test for differences in the rate of depressive and anxiety symptoms change (considered the clinical effect). These models compared the average symptom improvement rate in each cohort with the average symptom improvement rate in the Australian born cohort. Separate models were employed for each of the PHQ9 and GAD7 outcomes. All models specified a gamma scale and a log link function, to account for the proportion symptom change that occurs in outcome scales that are bounded at zero or minimal symptoms [28]. In addition, an unstructured working correlation matrix was specified to reflect different rates of change from pretreatment to posttreatment (i.e., the bulk of the therapeutic effect).

The statistical power of each longitudinal model was estimated with dedicated longitudinal modelling software [29] using the Australian born group as a reference model for estimating change over time, variance parameters and the within-subject correlation and associated with each outcome.

To explore the risk of adverse events amongst the groups, the individual rates of clinical change were also dichotomised into one of four classified outcome events used in the internet interventions [28, 30]; deterioration events (i.e., change that is > − 30% from baseline), non-response events (i.e., change between − 30% and + 30% from baseline), minimal response events (i.e., change that is 30 to 50% from baseline) or remission events (i.e., change that is > 50% from baseline). In this way, important clinical outcomes such as non-response to treatment or worsening of symptoms could be compared.

Together, the three sets of analyses aimed to plot the characteristics, progression and outcome of the treatment cohorts. All tests were conducted with a *p*-value of .01, given the small sample size in some cells. All analyses were performed using version 24 of the SPSS software.

#### Assessment of measurement bias

Possible sources of measurement bias were explored through the inclusion of three types of sensitivity analyses. First, the comparison of symptom change amongst the cohorts was tested with and without the effect of missing cases. This step was designed to account for impact of non-ignorable missing data following treatment. To account for missing cases each of analyses were conducted with the intention to treat principle, where the outcomes of participants who started treatment were included at post-treatment regardless of whether they had dropped out. Reflecting this principle, and in line with dedicated missing cases research in web-based psychotherapy trials [31], a Multiple Imputation (MI) procedure was employed, stratifying the estimation of missing cases for the following: (1) the rate of individual treatment dosage (estimated from the number of lessons downloaded); (2) the individual baseline symptom scores of each outcome; (3) the cohort membership; (4) symptom scores at each of the two time points; and (5) any two way interactions of these variables. To mitigate the effect of missing cases on binary outcomes, the series of logistic regressions were adjusted with a stratified bootstrap procedure, considering the above variables. Using the principle of intention to treat, the analyses that accounted for missing cases are considered as the primary analyses, with analyses that ignore missing cases considered as supplementary (i.e., labelled as Sensitivity Analysis 1 in Additional files 4 and 5).

Second, baseline differences between the migrant cohorts and the Australian-born cohort, that were significant (*p* < .05) and varied by greater than 10% from the Australian-born baseline were identified and included in a second set of sensitivity analyses. In this way, any demographic or clinical differences between the cohorts could be explored as potential confounds of the effect of migration (i.e., labelled as Sensitivity Analysis 2 in Additional files 4 and 5).

Third, the longitudinal models that estimate the symptom change of the five migrant cohorts were again modelled with the adjustment for the duration of residence in Australia. In this way, the adjusted models explored the effect of naturalisation as a possible moderator of migration influences and any difference between the cohorts (i.e., labelled as Sensitivity Analysis 3 in Additional files 4 and 5).

## Results

### Characteristics

There were significant differences between the six groups as shown in Fig. 2 and Table 1. The NESB migrants from Asia and the Middle East who started treatment tended to be more recent arrivals to Australia (< 10 years and < 5 years respectively) when compared to NESB Europe and NESB English groups. NESB migrants were younger, more likely to be male, were more likely to have university education, to live in an urban area, and reported lower mental health service use than those born in Australia. ESB migrants were more likely to be older, employed, have completed tertiary education and live in an urban area than those born in Australia. Finally, NESB English and ESB migrants who access MindSpot tended to have lived in Australia for longer (> 10 years).

The pre-treatment symptom scores for anxiety and depression scores were similar across groups. Migrants from NESB Asia, MidEast and Europe reported the highest rates of psychosocial difficulties in the domain of relationships. Migrants from NESB MidEast reported the lowest rates of difficulty in workplace/study and health when compared to all other groups. Migrants from NESB Asia reported the lowest rates of financial difficulty.

### Treatment outcomes

**Fig. 3:**
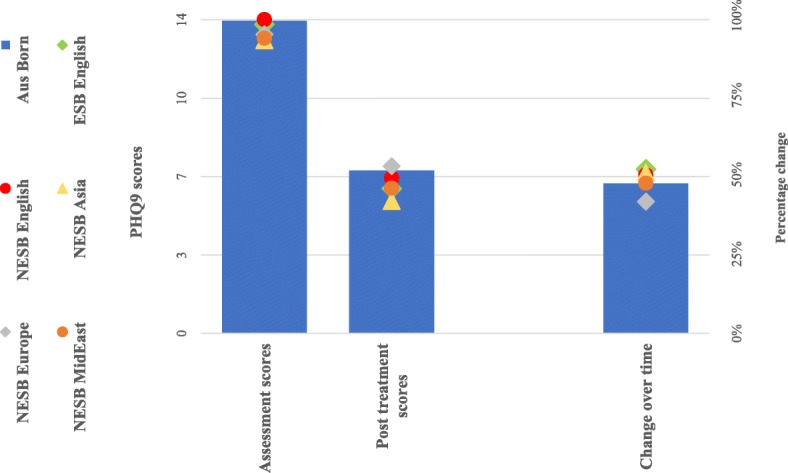
Depressive symptom scores and percentage change. Note. Aus = Australian; ESB = English Speaking Background; Europe = Europe region; MidEast = Middle Eastern or North African Region; NESB = Non-English Speaking Background; PHQ9 = Patient Health Questionnaire (9-item); Pre-Post = Pre-treatment to Post-treatment; Tx = Treatment; Δ = Change

**Fig. 4:**
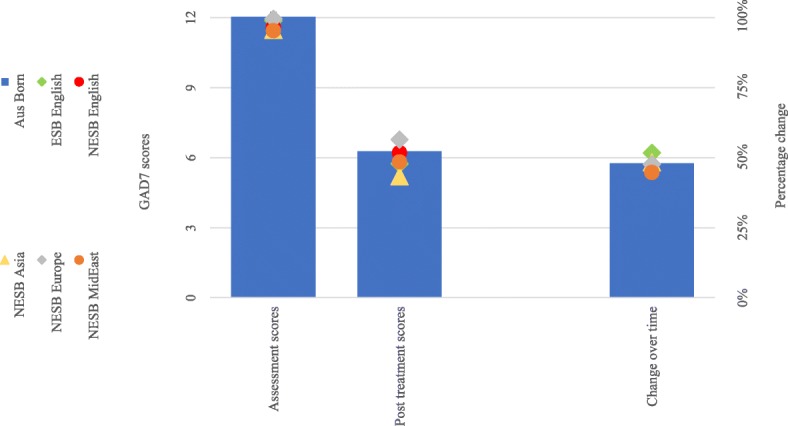
Anxiety symptom scores and percentage change**.** Note*.* Aus = Australian; ESB = English Speaking Background; Europe = Europe region; GAD7 = Generalised Anxiety Disorder Scale (7-item); MidEast = Middle Eastern or North African Region; NESB = Non-English Speaking Background; Pre-Post = Pre-treatment to Post-treatment; Tx = Treatment; Δ = Change

Results from the analyses of symptom change for each cohort are illustrated in Fig. 3 (depressive symptoms; PHQ9) and Fig. 4 (anxiety symptoms; GAD7). The complete numerical estimates and test statistics of effect sizes about the magnitude of symptom change are presented in Table 2. There was a 48% reduction in the average symptom scores in the Australian cohort.

**Table 2:** Post-treatment Change in Primary Outcomes

PHQ9	Pre EMM [95%CI]	Post EMM [95%CI]	∆%@ Pre-Post [95%CI]	Test of baseline scores differences (*p*-value)	Test of rate of change (*p*-value)	Achieved statistical power	Within group ES -Hedge’s g pre-post [95%CI]	Between group ES - Hedge’s g @post [95%CI]	Between group ∆% @ Pre-Post^a^ [95%CI]
NESB MidEast	12.88 [10.96, 14.81]	6.33 [4.47, 8.19]	51% [36, 65%]	.46	.68	20% (β = − 0.84)	1.13 [0.95, 1.32]	0.16 [0.04, 0.28]	11% [−20, 35%]
NESB Europe	13.05 [12.01, 14.1]	7.3 [6.28, 8.31]	44% [36, 52%]	.29	.38	20% (β = − 0.84)	1.08 [0.97, 1.2]	−0.03 [− 0.1, 0.05]	−2% [−19, 12%]
NESB Asia	12.79 [11.92, 13.67]	5.78 [4.72, 6.85]	55% [46, 63%]	.069	.12	40% (β = − 0.25)	1.35 [1.26, 1.45]	0.26 [0.2, 0.32]	19% [2, 33%]
NESB English	13.69 [13.06, 14.32]	6.87 [6.23, 7.51]	50% [45, 54%]	.88	.46	30% (β = −0.52)	1.28 [1.21, 1.35]	0.05 [0.01, 0.1]	4% [−8, 14%]
ESB English	13.48 [13.14, 13.83]	6.32 [6.0, 6.63]	53% [51, 56%]	.41	.001	90% (β = 1.28)	1.43 [1.39, 1.47]	0.15 [0.12, 0.18]	11% [6, 17%]
AusBorn	13.64 [13.49, 13.8]	7.13 [6.82, 7.43]	48% [46, 50%]	–	–	–	1.2 [1.18, 1.22]	–	–
GAD7	Pre EMM (95%CI)	Post EMM (95%CI)	∆%@ Pre-Post [95%CI]	Test of baseline scores differences (*p*-value)	Test of rate of change (*p*-value)	Achieved statistical power	Within group ES -Hedge’s g pre-post [95%CI]	Between group ES - Hedge’s g @post [95%CI]	Between group ∆% @ Pre-Post^a^ [95%CI]
NESB MidEast	11.44 [9.8, 13.09]	5.58 [3.16, 8]	51% [30, 72%]	.53	.071	20% (β = −0.84)	1.09 [0.91, 1.28]	0.16 [0.04, 0.28]	12% [−35, 42%]
NESB Europe	11.91 [10.95, 12.87]	6.75 [5.31, 8.19]	43% [31, 55%]	.88	.45	20% (β = −0.84)	1.07 [0.95, 1.18]	−0.11 [− 0.19, − 0.04]	−8% [−34, 14%]
NESB Asia	11.43 [10.68, 12.18]	5.18 [4.49, 5.87]	55% [49, 61%]	.16	.057	25% (β = − 0.68)	1.39 [1.3, 1.49]	0.25 [0.19, 0.31]	17% [4, 29%]
NESB English	11.55 [10.99, 12.11]	6.19 [5.56, 6.81]	46% [41, 52%]	.14	.59	30% (β = −0.52)	1.12 [1.06, 1.19]	0.02 [−0.03, 0.06]	1% [−8, 10%]
ESB English	11.83 [11.53, 12.13]	5.72 [5.45, 5.99]	52% [49, 54%]	.35	.006	90% (β = 1.28)	1.37 [1.33, 1.41]	0.12 [0.09, 0.15]	9% [4, 13%]
AusBorn	11.99 [11.86, 12.12]	6.26 [6.02, 6.5]	48% [46, 50%]	–	–	–	1.25 [1.23, 1.27]	–	–

Note. The multiple imputation procedure was adjusted for number of lesson completion (approximating treatment dose), time, Cohort group and all possible two way interaction between Dose, time and Cohort; ^a^ Reference Group is Ausborn; *Aus* Australian, *EMM* Estimated Marginal Means, *ESB* English Speaking Background, *Europe* Europe region, *GAD7* Generalised Anxiety Disorder Scale (7-item), *MI* Multiple Imputations, *MidEast* Middle Eastern or North Africa Region, *NESB* Non-English Speaking Background, *PHQ9* Patient Health Questionnaire (9-item), *Post* Post-treatment, *Pre* Pre-treatment, Δ Change

All four NESB cohorts achieved reductions in symptoms (i.e., 43 to 55%) comparable to the Australian born cohort at post treatment, whereas the reduction in symptom scores for both PHQ9 (53%) and GAD7 (52%) in the ESB cohort was better than for those born in Australia. Wider variation was observed in the NESB Asia groups (55%), but the results were not statistically significant because of the comparatively small sample size. The completion rates for the Australian-born cohort was an average of 3.86 lessons from a total of five lessons. Sixty-seven percent of Australian-born participants who started treatment completed all five lessons. Amongst the migrant groups, the rates of average lesson completion varied only slightly in comparison to the Australian-born cohort, and overall similar completion rates of participants were observed between the groups. Estimates and test statistics are presented in Table 1.

The achieved statistical power for rejecting false negatives (correctly determining no group differences) on both the anxiety and depression outcomes was low, as reported in Table 2 (Power: 20–90%; βpower range: − 0.84 – 1.28). This result is understandable because the symptoms outcome scores and the rate of symptom change within the five migrant cohort groups were very similar to the Australian born group. Given that the five migrant cohorts demonstrated effects that were within 87% of the effects observed in the Australian born group for both anxiety and depression, any residual clinical differences between the groups were considered marginal.

### Deterioration, non-response, minimal response and remission

**Fig. 5:**
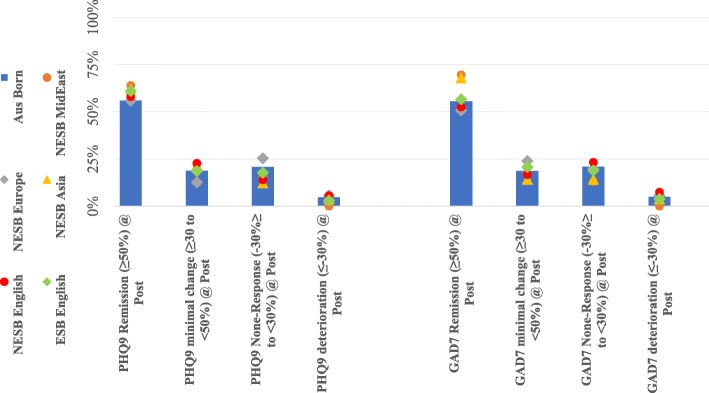
Proportions of individuals with four clinical categories**.***Note.* Aus = Australian; ESB = English Speaking Background; Europe = Europe region; GAD7 = Generalised Anxiety Disorder Scale (7-item); MidEast = Middle Eastern or North African Region; NESB = Non-English Speaking Background; PHQ9 = Patient Health Questionnaire (9-item); Post = Post-treatment

The estimated proportion of individuals who experienced either deterioration, non-response, minimal response or remission outcomes are shown in Fig. 5 and in Table 3. Fig. 5 shows that the majority of individual patients in each cohort had remission from symptoms (symptom reduction greater than 50% of baseline symptoms). A test of the probability of experiencing each of the outcomes between each of the cohorts demonstrated infrequent and minimal differences. For example, the ESB and NESB Asia cohorts were found to have a statistically significant increased rate of PHQ9 and GAD7 remission events respectively when compared to Australian-born group. Other findings include significantly reduced rates of PHQ9 non-response events for the NESB English cohort (14% of cases, compared to 21% in the Australian born group). Apart from those differences, the rates of remission, minimal response (symptom reductions of 30 to 50%), non-response (0–30% increase in symptoms, or 0–30% reductions), and deterioration events (increase in symptoms of over 30%) were similar between the groups.

**Table 3:** Deterioration, Non-response, Minimal Response and Remission Rates

	Bootstrapped PHQ9 categories of ∆@ Pre-Post		Nonresponse (−30 to 30%)	Deterioration (> − 30%)	Differences in proportions from AusBorn group (Bootstrapped *p*-values)			
	Remission (> 50%)	Minimal Response (30–50%)			Remission	Minimal Response	Nonresponse	Deterioration
NESB MidEast	64%	20%	16%	0%	.41	.88	.56	.99
NESB Europe	56%	13%	25%	6%	.92	.19	.35	.70
NESB Asia	62%	22%	12%	4%	.18	.46	.035	.67
NESB English	58%	23%	14%	6%	.58	.16	.017	.53
ESB English	61%	19%	18%	3%	.019	.89	.073	.061
Aus Born	56%	19%	21%	5%	–	–	–	–
	Bootstrapped GAD7 categories of ∆@ Pre-Post		Nonresponse	Deterioration	Differences in proportions from AusBorn group (Bootstrapped *p*-values)			
	Remission	Minimal Response			Remission	Minimal Response	Nonresponse	Deterioration
NESB MidEast	70%	17%	13%	0%	.18	.88	.36	.99
NESB Europe	51%	24%	20%	6%	.42	.26	.81	.79
NESB Asia	68%	14%	14%	4%	.012	.25	.093	.58
NESB English	53%	17%	23%	7%	.39	.50	.42	.11
ESB English	57%	21%	19%	4%	.61	.20	.25	.19
Aus Born	56%	19%	21%	5%	–	–	–	–

Note. Categories classified as deterioration (> − 30%), non-response (−30 to 30%), minimal response (30 to 50%) and remission (> 50%); *Aus* Australian, *ESB* English Speaking Background, *Europe* Europe region, *GAD7* Generalised Anxiety Disorder Scale (7-item), *MidEast* Middle Eastern or North African Region, *NESB* Non-English Speaking Background, *PHQ9* Patient Health Questionnaire (9-item), *Pre-Post* Pre-treatment to Post-treatment

### Sensitivity analyses and testing of confounds

In a precautionary step, the impact of missing cases, baseline differences between the cohorts and the duration of residence in Australia were analysed separately. Additional files 4, 5 and 6 illustrate the results of the primary analyses from each outcome of each cohort (in blue bars), in combination with the adjusted estimates outcome derived from the following sensitivity analyses overlaid with coloured error bars: (1) overlooking missing cases; (2) adjusting for baseline variables such as remoteness, gender, age, education and previous mental health service use; and (3) adjusting for the number of years since arrival in Australia.

The results presented in the sensitivity analyses (Additional files 4 and 5) show that overlooking missing cases can lead to lower post-treatment symptom scores and an overestimation of the clinical change at a margin that is consistent across all cohorts. The adjustment for baseline variables did not change the cohort trends or present new statistically significant findings. Similarly, adjusting for the number of years since migrating to Australia had only a small effect on the estimate of primary outcomes.

## Discussion

We found that 21.4% of the users of digital mental health service (DMHS) were born outside Australia, compared with 28% overseas born in the wider Australian community, and DMHS users came from 166 countries and territories, reflecting the diversity of the Australian population. The reported rate of past or current use of mental health services by migrants was lower than the Australian-born group, despite having symptoms of anxiety and depression that were similar in severity and duration, with the lowest rates among NESB migrants from Asia and Middle East. This is consistent with previous research that found NESB migrants in Australia are less likely to access mental health services [32].

The lower proportion of migrants accessing the DMHS, MindSpot, might be due to reluctance by some migrants to seek treatment, even free and anonymous online treatment, or less awareness of the presence of mental disorders and the availability of effective treatment. However, it might also reflect the lower rate of depression and anxiety among migrants found by the National Mental Health and Well Being surveys. The finding that the ratio of service users of ESB and NESB was similar to that found in the Australian population might reflect the higher level of education and command of written English of NESB migrants. Both NESB and ESB migrants were more likely to have completed tertiary education, which is not surprising because nearly two thirds of recent migrants arrived as skilled migrants [33].

NESB migrants were younger and more likely to live in an urban area, a finding that is consistent with official data regarding the age and distribution of the migrant population of Australia respectively [2]. The higher proportion of males among NESB migrant users might be due to the anonymity of online treatment, protecting individual and family reputation, as psychological treatment among people from traditional societies carry a particular stigma and might affect family reputation [34].

Migrants across all groups generally reported lower rates of psychosocial difficulties than Australian-born users, possibly because migrants seeking treatment at the DMHS had better education and more opportunities for employment as a result of coming to Australian on the basis of their education and skills.

With regards to treatment outcome, migrant users who went on to complete the online treatment obtained reductions in symptoms of anxiety and depression equivalent to those born in Australia. The rates of remission in all migrant groups, including rates of minimal response (symptom reductions of 30 to 50%), non-response (0–30% increase in symptoms, or 0–30% reductions), and deterioration events (increase in symptoms of over 30%) were similar to each other and to those born in Australia. This is again not entirely surprising, as the treatments have been developed over several years with feedback from large numbers of research participants, many of whom were also migrants. Treatment outcomes for NESB and ESB migrants were similar to those of Australian-born patients, both in terms of the rate of completion of courses and in the reduction in symptoms. Migrant users who enrol in treatment generally benefit from treatment, however there were not many migrants from NESB countries who did not speak English as the main language at home. For example, only 43 patients from the Middle East (0.6%) and 182 from Asian region (2.7%) started treatment over the three-year period of this current study, which is lower than the Australian populations of these regional language groups, that is, Arabic (1.4%) and Cantonese/Mandarin (3.9%) [35]. This pattern of low uptake, despite the effectiveness of the treatment in these groups is consistent with the limited research of internet-delivered treatment conducted with migrant populations in Australia [10], and may indicate a need for digital mental health services to be provided in languages other than English as well the as promotion of DMHS targeting relevant ethnic groups. This need is reflected in the Productivity Commission’s recommendation of expansion of online mental health treatment to cater for culturally and linguistically diverse groups.

Overall, the results of the present study are consistent with previous research which found DMHS have the potential to overcome barriers to care and improve mental health outcomes for migrants [36]. DMHS have the potential to overcome the barriers of limited command of English and low income by offering the courses in languages other than English free of charge, which is currently underway. However, a greater challenge in many migrant communities is the low level of mental health literacy, and distrust of mental health services. Research into methods of improving mental health literacy and acceptance of treatment in migrants communities is needed. For example, developing digital mental health services and online resources that are championed by religious and cultural leaders, and delivered within local migrant communities may help to increase acceptance and mental health literacy. Despite these challenges, the results demonstrate that internet-delivered cognitive behaviour therapy (iCBT) can be effective for people from a range of cultural backgrounds.

A limitation of these findings is that the information about migrant status and most of the other data including symptom scores was self-reported. However, the sample size for most migrant groups was relatively large, and most of the patients were contacted by telephone during assessment and treatment, in many cases confirming the country of origin. Moreover, the results are similar to those of clinical trials in which patients were more closely followed up. Another limitation is the high proportion of migrant users who reported university level education, and although the courses are equally effective in Australian born users with varying levels of education, the courses may not be as effective in migrants with lower levels of education, who are also likely to have less proficiency in English. A further limitation is that we were unable to consider the influence of Australian-born users who were brought up in migrant families, in which the main language spoken at home is often the language spoken by migrant parents. However, Australians brought up in non-English speaking families generally had the benefit of attending a minimum of 10 years of school with instruction in English, and would be considered as native speakers.

## Conclusions

With those limitations in mind this study supports the potential of emerging online treatments for treating anxiety and depression in both NESB and ESB migrants and confirms the potential of DMHS to improve mental health outcomes and reduce barriers to care for migrants to Australia and to other countries. DMHS, which are provided by trained therapists operating within an established clinical governance framework, should be seen as a treatment option alongside face to face mental health services, including those specifically for migrants.

## Supplementary information

**Additional file 1.** Country of Birth and Migration Status Allocation. Table denoting participant’s country of origin. Migrants were further classified into grouping that represent migrants from an English-speaking background (ESB) or non-English-Speaking background (NESB).

**Additional file 2.** Classification of Groups by Language Regions. This table denotes the classification of participant groups based on main language spoken at home and divided into the following five language region groups; (1) English, (2) South or East Asian language, (3) Arabic or other Middle-Eastern language, (4) a European language or (5) other than the above

**Additional file 3.** Final Sample Sizes of Cohorts in Treatment. This table denotes the classification of participants based on country of origin (i.e., Australian-born vs. migrant) and regional language spoken at home to form the six distinct groups of online treatment users.

**Additional file 4:.** Sensitivity Analyses of Depression (PHQ9) Outcomes. This figure shows a sensitivity analysis adjusting for ignoring missing cases, baseline variables such as remoteness, gender, age, education and previous mental health service use, and years since arriving in Australia (i.e., years naturalised) on depression outcomes.

**Additional file 5.** Sensitivity Analyses of Anxiety (GAD7) Outcomes. This figure shows a sensitivity analysis adjusting for ignoring missing cases, baseline variables such as remoteness, gender, age, education and previous mental health service use, and years since arriving in Australia (i.e., years naturalised) on depression outcomes.

**Additional file 6.** Sensitivity Analyses for PHQ9 and GAD7. This table shows a sensitivity analysis adjusting for ignoring missing cases, baseline variables such as remoteness, gender, age, education and previous mental health service use, and years since arriving in Australia (i.e., years naturalised) on primary treatment outcomes.

## Abbreviations

AusBorn: People Born In Australia
DMHS: Digital Mental Health Service
ESB: English Speaking Background
GAD7: Generalized Anxiety Disorder Scale – 7 Item
iCBT: Internet-delivered Cognitive Behavioural Therapy
MidEast: Middle East or North Africa language regions
NESB: Non-English Speaking Background
PHQ-9: Patient Health Questionnaire-9 Item
