# Supplementary material for: A comparison of the characteristics and treatment outcomes of migrant and Australian-born users of a national digital mental health service
Source: BMC Psychiatry. 2020 Mar 11;20:111. doi: 10.1186/s12888-020-02486-3 (PMC7065305; doi:10.1186/s12888-020-02486-3)
Supplement: Supplementary file 1 — Additional file 1. Country of Birth and Migration Status Allocation. Table denoting participant’s country of origin. Migrants were further classified into grouping that represent migrants from an English-speaking background (ESB) or non-English-Speaking background (NESB). [file 12888_2020_2486_MOESM1_ESM.docx]

Additional File 2 - Classification of Groups by Language Regions

This table denotes the classification of participant groups based on main language spoken at home and divided into the following five language region groups; (1) English, (2) South or East Asian language, (3) Arabic or other Middle-Eastern language, (4) a European language or (5) other than the above

| **Language** | **Language regions** | |  |  |  |  |
| --- | --- | --- | --- | --- | --- | --- |
|  | **English** | **Asia** | **European** | **MidEast** | **Other** |  |
| **English** | 6303 | 0 | 0 | 0 | 0 |  |
| **Mandarin** | 0 | 33 | 0 | 0 | 0 |  |
| **Hindi** | 0 | 30 | 0 | 0 | 0 |  |
| **Cantonese** | 0 | 29 | 0 | 0 | 0 |  |
| **Vietnamese** | 0 | 14 | 0 | 0 | 0 |  |
| **Urdu** | 0 | 13 | 0 | 0 | 0 |  |
| **Tamil** | 0 | 11 | 0 | 0 | 0 |  |
| **Chinese** | 0 | 10 | 0 | 0 | 0 |  |
| **Nepali** | 0 | 9 | 0 | 0 | 0 |  |
| **Indonesian** | 0 | 6 | 0 | 0 | 0 |  |
| **Korean** | 0 | 6 | 0 | 0 | 0 |  |
| **Japanese** | 0 | 5 | 0 | 0 | 0 |  |
| **Kannada** | 0 | 5 | 0 | 0 | 0 |  |
| **Punjabi** | 0 | 5 | 0 | 0 | 0 |  |
| **Tagalog** | 0 | 5 | 0 | 0 | 0 |  |
| **Bengali** | 0 | 4 | 0 | 0 | 0 |  |
| **Filipino** | 0 | 4 | 0 | 0 | 0 |  |
| **Hokkien** | 0 | 3 | 0 | 0 | 0 |  |
| **Malay** | 0 | 3 | 0 | 0 | 0 |  |
| **Malayalam** | 0 | 3 | 0 | 0 | 0 |  |
| **Singhalese** | 0 | 3 | 0 | 0 | 0 |  |
| **Bengali (Bangla)** | 0 | 2 | 0 | 0 | 0 |  |
| **Burmese** | 0 | 2 | 0 | 0 | 0 |  |
| **Lao** | 0 | 2 | 0 | 0 | 0 |  |
| **Sinhaleese** | 0 | 2 | 0 | 0 | 0 |  |
| **Sinhalese** | 0 | 2 | 0 | 0 | 0 |  |
| **Bahasa Malaysia** | 0 | 1 | 0 | 0 | 0 |  |
| **Bangla** | 0 | 1 | 0 | 0 | 0 |  |
| **Chinese and Vietnamese** | 0 | 1 | 0 | 0 | 0 |  |
| **English and Japanese** | 0 | 1 | 0 | 0 | 0 |  |
| **English, cantonese** | 0 | 1 | 0 | 0 | 0 |  |
| **Filipino, English** | 0 | 1 | 0 | 0 | 0 |  |
| **Gujarati** | 0 | 1 | 0 | 0 | 0 |  |
| **Sinhala** | 0 | 1 | 0 | 0 | 0 |  |
| **Telugu** | 0 | 1 | 0 | 0 | 0 |  |
| **Thai/ English** | 0 | 1 | 0 | 0 | 0 |  |
| **Urdu/Hindi** | 0 | 1 | 0 | 0 | 0 |  |
| **Spanish** | 0 | 0 | 35 | 0 | 0 |  |
| **Greek** | 0 | 0 | 23 | 0 | 0 |  |
| **Italian** | 0 | 0 | 20 | 0 | 0 |  |
| **German** | 0 | 0 | 16 | 0 | 0 |  |
| **Portuguese** | 0 | 0 | 14 | 0 | 0 |  |
| **Russian** | 0 | 0 | 12 | 0 | 0 |  |
| **French** | 0 | 0 | 11 | 0 | 0 |  |
| **Macedonian** | 0 | 0 | 9 | 0 | 0 |  |
| **Polish** | 0 | 0 | 9 | 0 | 0 |  |
| **Serbian** | 0 | 0 | 9 | 0 | 0 |  |
| **Dutch** | 0 | 0 | 5 | 0 | 0 |  |
| **Armenian** | 0 | 0 | 2 | 0 | 0 |  |
| **Croatian** | 0 | 0 | 2 | 0 | 0 |  |
| **Finnish** | 0 | 0 | 2 | 0 | 0 |  |
| **Hungarian** | 0 | 0 | 2 | 0 | 0 |  |
| **Romanian** | 0 | 0 | 2 | 0 | 0 |  |
| **Albanian** | 0 | 0 | 1 | 0 | 0 |  |
| **Bosnian** | 0 | 0 | 1 | 0 | 0 |  |
| **Czech Language** | 0 | 0 | 1 | 0 | 0 |  |
| **Dansih** | 0 | 0 | 1 | 0 | 0 |  |
| **English and Dutch** | 0 | 0 | 1 | 0 | 0 |  |
| **English and Serbian** | 0 | 0 | 1 | 0 | 0 |  |
| **English, Finnish** | 0 | 0 | 1 | 0 | 0 |  |
| **English/Armenian** | 0 | 0 | 1 | 0 | 0 |  |
| **English/Russian** | 0 | 0 | 1 | 0 | 0 |  |
| **French/english** | 0 | 0 | 1 | 0 | 0 |  |
| **Greek/English** | 0 | 0 | 1 | 0 | 0 |  |
| **Maltese** | 0 | 0 | 1 | 0 | 0 |  |
| **Mied English + Spanish** | 0 | 0 | 1 | 0 | 0 |  |
| **russian** | 0 | 0 | 1 | 0 | 0 |  |
| **serbian/bosnian** | 0 | 0 | 1 | 0 | 0 |  |
| **Slovenian** | 0 | 0 | 1 | 0 | 0 |  |
| **spanish/english** | 0 | 0 | 1 | 0 | 0 |  |
| **Arabic** | 0 | 0 | 0 | 33 | 0 |  |
| **Farsi** | 0 | 0 | 0 | 14 | 0 |  |
| **Persian** | 0 | 0 | 0 | 6 | 0 |  |
| **Turkish** | 0 | 0 | 0 | 5 | 0 |  |
| **Arabic and English** | 0 | 0 | 0 | 1 | 0 |  |
| **english & arabic** | 0 | 0 | 0 | 1 | 0 |  |
| **english and turkish** | 0 | 0 | 0 | 1 | 0 |  |
| **Persian/English** | 0 | 0 | 0 | 1 | 0 |  |
| **Afrikaans** | 0 | 0 | 0 | 0 | 13 |  |
| **Auslan** | 0 | 0 | 0 | 0 | 4 |  |
| **Maori** | 0 | 0 | 0 | 0 | 3 |  |
| **Shona** | 0 | 0 | 0 | 0 | 2 |  |
| **Creole** | 0 | 0 | 0 | 0 | 1 |  |
| **Fijian** | 0 | 0 | 0 | 0 | 1 |  |
| **Hebrew** | 0 | 0 | 0 | 0 | 1 |  |
| **Khmer** | 0 | 0 | 0 | 0 | 1 |  |
| **Pashto** | 0 | 0 | 0 | 0 | 1 |  |
| **Samoan** | 0 | 0 | 0 | 0 | 1 |  |
| **Telegu** | 0 | 0 | 0 | 0 | 1 |  |
| **Teochew** | 0 | 0 | 0 | 0 | 1 |  |
| **Visayan** | 0 | 0 | 0 | 0 | 1 |  |
|  |  |  |  |  |  |  |
| **Total** | 6303 | 222 | 190 | 62 | 31 | 6808 |
|  |  |  |  |  |  |  |
| **Percentage** | 92.6% | 3.3% | 2.8% | 0.9% | 0.5% | 100.0% |
