# Supplementary material for: A comparison of the characteristics and treatment outcomes of migrant and Australian-born users of a national digital mental health service
Source: BMC Psychiatry. 2020 Mar 11;20:111. doi: 10.1186/s12888-020-02486-3 (PMC7065305; doi:10.1186/s12888-020-02486-3)
Supplement: Supplementary file 3 — Additional file 3. Final Sample Sizes of Cohorts in Treatment. This table denotes the classification of participants based on country of origin (i.e., Australian-born vs. migrant) and regional language spoken at home to form the six distinct groups of online treatment users. [file 12888_2020_2486_MOESM3_ESM.docx]

Additional File 4 - Sensitivity Analyses of Depression (PHQ9) Outcomes

This figure shows a sensitivity analysis adjusting for ignoring missing cases, baseline variables such as remoteness, gender, age, education and previous mental health service use, and years since arriving in Australia (i.e., years naturalised) on depression outcomes.

*
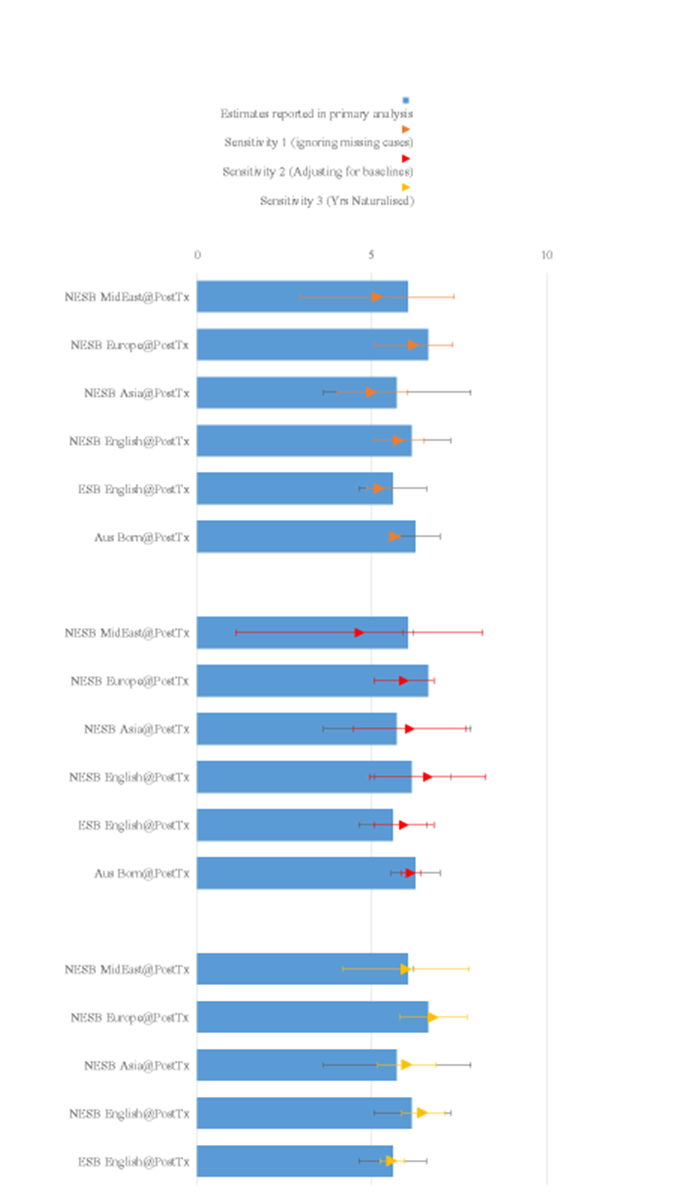
*

*Note.* Aus = Australian; ESB = English Speaking Background; Euro = Europe region; MI= Multiple Imputation; MidEast = Middle Eastern Region; NESB = Non-English Speaking Background; PHQ9 = Patient Health Questionnaire (9-item); PreTx = Pre-treatment; Post = Post-treatment; yrs = years.
