# Supplementary material for: A comparison of the characteristics and treatment outcomes of migrant and Australian-born users of a national digital mental health service
Source: BMC Psychiatry. 2020 Mar 11;20:111. doi: 10.1186/s12888-020-02486-3 (PMC7065305; doi:10.1186/s12888-020-02486-3)
Supplement: Supplementary file 5 — Additional file 5. Sensitivity Analyses of Anxiety (GAD7) Outcomes. This figure shows a sensitivity analysis adjusting for ignoring missing cases, baseline variables such as remoteness, gender, age, education and previous mental health service use, and years since arriving in Australia (i.e., years naturalised) on depression outcomes. [file 12888_2020_2486_MOESM5_ESM.docx]

Additional File 6

Sensitivity Analyses for PHQ9 and GAD7

This table shows a sensitivity analysis adjusting for ignoring missing cases, baseline variables such as remoteness, gender, age, education and previous mental health service use, and years since arriving in Australia (i.e., years naturalised) on primary treatment outcomes.

|  | **PHQ9 (Depression) Estimates reported in primary analyses** | **p-value of the primary analysis** | **Sensitivity 1 (analysis ignoring missing cases)** | **p-value of the sensitivity analysis** |
| --- | --- | --- | --- | --- |
|  |  |  |  |  |
| **NESB MidEast@PostTx** | 6.15 (1) | 0.832 | 5.52 (1) | 0.154 |
| **NESB Eaurope@PostTx** | 7.73 (0.7) | 0.484 | 6.6 (0.6) | 0.663 |
| **NESB Asia@PostTx** | 6.08 (0.5) | 0.408 | 5.23 (0.6) | 0.310 |
| **NESB English@PostTx** | 6.7 (0.4) | 0.484 | 6.1 (0.4) | 0.125 |
| **ESB English@PostTx** | 6.22 (0.3) | 0.071 | 5.65 (0.2) | 0.114 |
| **Aus Born@PostTx** | 7.11 (0.1) |  | 6.36 (0.1) |  |
|  |  |  |  |  |
|  |  |  | **Sensitivity 2 (Analysis adjusting for baseline covariates)** |  |
| **NESB MidEast@PostTx** | 6.15 (1) | 0.832 | 5.54 (1.6) | 0.288 |
| **NESB Eaurope@PostTx** | 7.73 (0.7) | 0.484 | 6.69 (0.4) | 0.265 |
| **NESB Asia@PostTx** | 6.08 (0.5) | 0.408 | 6.47 (0.8) | 0.925 |
| **NESB English@PostTx** | 6.7 (0.4) | 0.484 | 7.43 (0.8) | 0.949 |
| **ESB English@PostTx** | 6.22 (0.3) | 0.071 | 6.69 (0.4) | 0.528 |
| **Aus Born@PostTx** | 7.11 (0.1) |  | 6.98 (0.1) |  |
|  |  |  |  |  |
|  |  |  | **Sensitivity 3 (analysis ajusting for years naturalised)** |  |
| **NESB MidEast@PostTx** | 6.15 (1) | -- | 6.11 (0.9) | -- |
| **NESB Eaurope@PostTx** | 7.73 (0.7) | -- | 8.61 (0.6) | -- |
| **NESB Asia@PostTx** | 6.08 (0.5) | -- | 6.75 (0.5) | -- |
| **NESB English@PostTx** | 6.7 (0.4) | -- | 7.01 (0.3) | -- |
| **ESB English@PostTx** | 6.22 (0.3) | -- | 6.42 (0.2) | -- |
| **Aus Born@PostTx** | 7.11 (0.1) |  |  |  |
|  |  |  |  |  |
|  | **GAD7 (Anxiety) Estimates reported in primary analysis** | **p-value of the primary analysis** | **Sensitivity 1 (ignoring missing cases)** | **p-value of the sensitivity analysis** |
| **NESB MidEast@PostTx** | 6.05 (1.1) | 0.502 | 5.17 (1.1) | 0.026 |
| **NESB Eaurope@PostTx** | 6.63 (0.6) | 0.662 | 6.21 (0.6) | 0.459 |
| **NESB Asia@PostTx** | 5.73 (0.5) | 0.182 | 5.01 (0.5) | 0.035 |
| **NESB English@PostTx** | 6.16 (0.4) | 0.312 | 5.77 (0.4) | 0.450 |
| **ESB English@PostTx** | 5.61 (0.2) | 0.002 | 5.21 (0.2) | 0.152 |
| **Aus Born@PostTx** | 6.25 (0.1) |  | 5.67 (0.1) |  |
|  |  |  |  |  |
|  |  |  | **Sensitivity 2 (Adjusting for baselines)** |  |
| **NESB MidEast@PostTx** | 6.05 (1.1) | 0.502 | 4.65 (1.8) | 0.704 |
| **NESB Eaurope@PostTx** | 6.63 (0.6) | 0.662 | 5.92 (0.4) | 0.169 |
| **NESB Asia@PostTx** | 5.73 (0.5) | 0.182 | 6.1 (0.8) | 0.971 |
| **NESB English@PostTx** | 6.16 (0.4) | 0.312 | 6.6 (0.8) | 0.726 |
| **ESB English@PostTx** | 5.61 (0.2) | 0.002 | 5.92 (0.4) | 0.488 |
| **Aus Born@PostTx** | 6.25 (0.1) |  | 6.12 (0.1) |  |
|  |  |  |  |  |
|  |  |  | **Sensitivity 3 (Yrs Naturalised)** |  |
| **NESB MidEast@PostTx** | 6.05 (1.1) | -- | 5.98 (0.9) | -- |
| **NESB Eaurope@PostTx** | 6.63 (0.6) | -- | 6.77 (0.5) | -- |
| **NESB Asia@PostTx** | 5.73 (0.5) | -- | 6.02 (0.4) | -- |
| **NESB English@PostTx** | 6.16 (0.4) | -- | 6.47 (0.3) | -- |
| **ESB English@PostTx** | 5.61 (0.2) | -- | 5.59 (0.2) | -- |
| **Aus Born@PostTx** | 6.25 (0.1) |  |  |  |
|  |  |  |  |  |
|  |  |  |  |  |

*Note*. Aus = Australian; ESB = English Speaking Background; Euro = Europe region; MI= Multiple Imputation; MidEast = Middle Eastern Region; NESB = Non-English Speaking Background-; PreTx = Pre-treatment; Post = Post-treatment; yrs = years.
